# A Business Girl's Views on the Slum Troubles

**Published:** 1919-08-09

**Authors:** 


### SOME PRESENT-DAY LIFE CONDITIONS.

### A Business Girl's Views on the Slum Troubles.

MUCH has recently been written regarding the slum problem. I am not a slum worker, but just an ordinary business girl who has to pass along several slum streets to and from my place of employment; and, as I have been passing through them sometimes twice and often four times a day for nearly ten years, I sometimes hear a little concerning the inhabitants.

A few weeks ago I heard through a plumber, who gets repeatedly called in to do repairs, that a certain family had taken one of the few really decent, well-built houses in the neighbourhood, a house that was probably originally built for a manager of one of the surrounding factories. After moving into the house, these people let every available room, and altogether there were about a dozen children living there. The house contained a very nice bathroom and lavatory combined; but, instead of being the Godsend it should have been to them, what happened? All the children are in the habit of playing in the bathroom. They sit on the sides of the bath, with their feet dangling inside, pretending they are in an omnibus, whilst one child acts as conductor of the imaginary 'bus, standing on the lavatory seat and pulling the chain, once to stop the 'bus, twice to start it again, and three times when the supposed 'bus is full up. All kinds of old tram and 'bus tickets, collected from the streets, are used, and then either pushed down the lavatory or left in the bath; and, needless to say, the once "nice" bathroom is now a perfect disgrace. The landlord cannot turn the tenants out, and, with the present labour troubles, it is both difficult and expensive to keep the house in anything like a sanitary condition. It would not matter what kind of house such folks lived in, for they do not want to live decently.

Really, I consider that lack of suitable education is responsible for many of the slum troubles. Knock out some of the unnecessary subjects that are taught in the Public Elementary Schools, leaving them to be taught to children who are brilliant enough to pass on to the Secondary Schools. Teach the three R's, and, above all, teach hygiene, commencing as early as possible; and, later on, teach elementary physiology. For a long time—ever since the formation of the National Council for Combating Venereal Diseases (with which my business has caused me to have some dealings)—I have felt that the only satisfactory, although somewhat slow, way of stamping out venereal disease, and also of developing our race into an A1 race, is by teaching physiology in our public elementary schools.

This is somewhat wandering away from the "Slum" question. Returning to that, I think that if it were possible to connect with each school a simply furnished house, which the children could take turns in cleaning and attending to ventilation, etc., the idea of a sanitary dwelling would be instilled in their minds, so that by the time they were old enough to leave school they would either reform their parents or leave the terrible homes many of them are brought up in. Hygiene lessons could be given at the house by a qualified teacher. I am convinced that until people understand how to look after and appreciate a decent abode we shall never get rid of the slums. There seems little use in expending huge sums of money on building nice cottages, etc., for people such as I have mentioned above, who will soon turn them into insanitary hovels unfit for human occupation. Of course, much improvement needs to be made, and made immediately; but until the ideas of common cleanliness are driven home to many of the people the slum trouble will keep recurring. Much might be done by having adult schools, but the class of people who need them most would not attend them unless compelled to do so. Hundreds of the folks do not want to be clean. I met a great specialist in town some weeks ago, and spoke to him on the same subject, and he confirmed my idea, that there are quite a number of persons who prefer to be dirty and live in dirt than to be clean; for he says that it is simply disgusting that people should attend our large hospitals in the filthy state many of them do. They know they are going, but do not even take the trouble to wash themselves before doing so.

Not being a writer, I find it rather difficult to express myself clearly; but I trust that if my views are put into print, some thoughtful and intelligent person will read them and grasp my full meaning (which is not any way calculated to run the poor folks down); and perhaps will be helped to do something that will be of real benefit to those poor slum dwellers who need help, and, incidentally, our race to become A1.

